# Supplementary material for: Integration and multiplexing of positional and contextual information by the hippocampal network
Source: PLoS Comput Biol. 2018 Aug 14;14(8):e1006320. doi: 10.1371/journal.pcbi.1006320 (PMC6117099; doi:10.1371/journal.pcbi.1006320)
Supplement: S1 Text — Effective two-state model for hippocampal CANN activityEffects of parameters on the model propertiesRelationship between sojourn time and correlation timeInference of path-integrator realignment times: discussion on parameters p0 and peIndependence of frequency of flickers from delay after light switch: parameters p0, pe, and L0Assessment of performances of map decoderDependence of positional-error analysis with L0. (PDF) [file pcbi.1006320.s001.pdf]

Integration and multiplexing of positional and contextual information by the hippocampal network: S1 Text

Lorenzo Posani[1*], Simona Cocco[1*], and Rémi Monasson[2*]

[1]Laboratory of Statistical Physics, Ecole Normale Supérieure and CNRS UMR 8550, PSL Research, Paris Sorbonne UPMC, 24 rue Lhomond, 75005 Paris, France
[2]Laboratory of Theoretical Physics, Ecole Normale Supérieure and CNRS UMR 8549, PSL Research, Paris Sorbonne UPMC, 24 rue Lhomond, 75005 Paris, France.

*email: lorenzo.posani@gmail.com (LP); cocco@lps.ens.fr (SC); monasson@lpt.ens.fr (RM)

Contents

1 Effective two-state model for hippocampal CANN activity **2**

2 Effects of parameters on the model properties **4**

3 Relationship between sojourn time and correlation time **7**

4 Inference of path-integrator realignment times - discussion on parameters p_0 and p_e **8**

5 Independence of frequency of flickers from delay after light switch: parameters p_0 and p_e and L_0 **9**

6 Assessment of performances of map decoder **11**

7 Dependence of positional-error analysis with L_0 **11**

1 Effective two-state model for hippocampal CANN activity

We show below how the two-state model pictured in Main Text, Fig. 3B, can be derived from the definition of the microscopic CANN model, see Main Text, Methods. The dynamical evolution of the CANN ensures that the log probability of a configuration of activity $\mathbf{s} = \{s_i\}$ is given by [1]

$$L(\mathbf{s}) = \sum_{i<j} J_{ij}\, s_i\, s_j + \sum_i \big(h_i^V(\mathbf{r}) + h_i^{PI}(\mathbf{r})\big)\, s_i + L^* \ , \tag{1}$$

where $\mathbf{r}$ if the rodent position and L^* is a constant term such that the sum of the probabilities e^L over all 2^N configurations $\mathbf{s}$ is normalized to unity.

For simplicity, we consider that the bump of activity consists of $a \times N$ active neurons $s_i = 1$ with place-field centers as close as possible in a map, say, $m = A$, where a is the fraction of active neurons in any time bin. This corresponds to the limiting case of zero neural noise, $\beta \to \infty$ [2]; calculation of effective potentials at finite β is much more involved and requires the use of sophisticated statistical physics techniques able to take into account the fluctuations of neural activities, see [3].

Let us define r_{bump} as the radius of the bump, i.e. the maximal distance in environment m between the rodent position $\mathbf{r}$ and the place-field centers r_i^m of active neurons. We have

$$a\, N = \frac{\pi\, r_{max}^2}{\delta^2} \ , \tag{2}$$

where δ^2 is the elementary portion of surface per place cell, defined as the total area of the environment, L^2, over the number of place cells, N. We thus obtain the expression of the bump radius as a function of the activity,

$$r_{max}(a) = L\sqrt{\frac{a}{\pi}} \ . \tag{3}$$

Contributions to log-likelihood due to inputs.

We assume that the CANN has activity localized in map m, and that the whole system is in the conflicting phase, with $PI = A$ and $V = B$. The contributions to L due to the visual (V) and path-integrator (PI) inputs reads, up to quadratic terms in a,

$$L_{input} = \gamma \sum_i s_i\, \phi\big(\mathbf{r}_i^M - \mathbf{r}\big) \ , \tag{4}$$

where $\gamma = \gamma_{PI}$ if $m = A$ and $\gamma = \gamma_V$ if $m = B$. According to the definition of the bump radius r_{max}, we have

$$L_{input} = \gamma \int_0^{r_{max}} \frac{d\mathbf{r}}{\delta^2}\, \phi(\mathbf{r}) = \frac{\gamma}{\sigma^2} \int_0^{r_{max}} dr\, r\, e^{-r^2/(2\sigma^2)} = \gamma\left(1 - e^{-r_{max}^2/(2\sigma^2)}\right) \ . \tag{5}$$

Contributions to log-likelihood due to recurrent connections.

We now consider the contribution $L_{recurrent}$ to the log-likelihood coming from the recurrent connection in the CANN. The coupling J_{ij} between neurons i and j is the sum of one interaction specific to map A and another one specific to map B, see Eqn. (9) in Main Text, Methods. Assuming again that the bump of activity is localized in map $m = A$, we neglect the contribution to L due to the

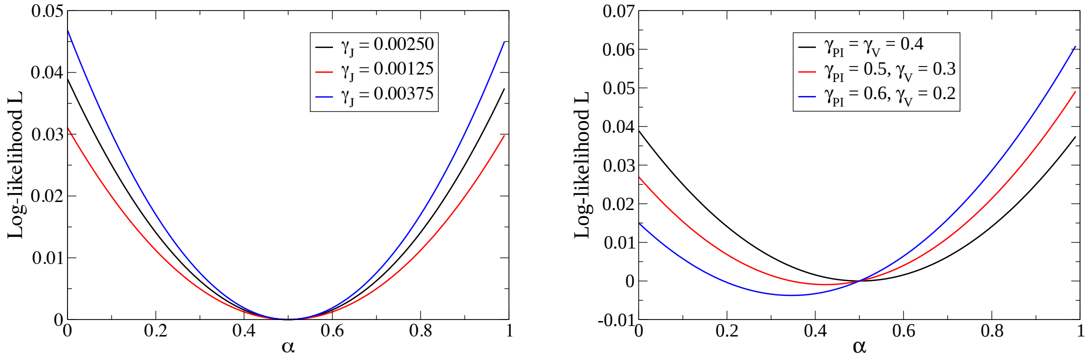

Supplementary Figure A. Log-likelihood of a mixed state with a fraction α of bump in state A and a fraction $1-\alpha$ in state B. An additive constant, independent of α, is introduced such that $L=0$ for $\alpha = 0.5$. Top: symmetric case $\gamma_{PI} = \gamma_V = 0.4$ for three values of γ_J, showing how the intensity of recurrent connections control the depth in log-likelihood of the mixed state. Bottom: Asymmetric case with $\gamma_J = 0.0025$ and for three values of $\gamma_{PI} > \gamma_V$. In all cases, $\sigma/L = 0.125$, $a = 0.1$.

interaction specific to map B. This simplifying approximation amounts to an error of the order of a^2, see [2] for more details. We obtain

$$\begin{aligned} L_{recurrent} &= \frac{1}{2}\,\gamma_J \int_0^{r_{max}} \frac{d\mathbf{r}}{\delta^2} \int_0^{r_{max}} \frac{d\mathbf{r}'}{\delta^2}\,\phi\big(\mathbf{r}-\mathbf{r}'\big) = \frac{\gamma_J\,N}{4\pi\,L^2\,\sigma^2} \int_0^{r_{max}} \int_0^{r_{max}} d\mathbf{r}\,d\mathbf{r}'\,e^{-(\mathbf{r}-\mathbf{r}')^2/(2\sigma^2)} \\ &= \gamma_J\,\frac{2\pi\,N\,\sigma^2}{L^2} \int_0^{\left(\frac{r_{max}}{\sigma}\right)^2} du\,I_0(u) \int_u^{\left(\frac{r_{max}}{\sigma}\right)^2} dv\,\frac{e^{-v}}{\sqrt{\left(\frac{v}{u}\right)^2 - 1}}\,, \end{aligned} \tag{6}$$

where I_0 is the first kind modified Bessel function of zero order.

Case of mixed state.

Assume now that a fraction α of the bump is localized in map $m = A$ and the remaining fraction, $1-\alpha$, is localized in map B. The log-likelihood of this mixed state is obtained by summing the expressions of the log-likelihood in state A above with activity $a \to \alpha\,a$ and of the log-likelihood in state B above with activity $a \to (1-\alpha)\,a$. The result is shown in Supplementary Fig. A. As indicated in Main Text, the amplitude γ_J of the recurrent connections controls the depth of the well separating the two complete bump states (all A or all B), while the ratio γ_{PI}/γ_V controls the asymmetry of the log-likelihood profile and favors one of the two states.

2 Effects of parameters on the model properties

The CANN model is defined up to a set of parameters:

(a) the level of neural noise in the simulated activity, β; higher β corresponding to lower noise. This parameter is formally equivalent to the inverse temperature in the Monte Carlo simulation;

(b) the strength of the recurrent connectivity, γ_J;

(c) the strength of the two inputs, γ_V and γ_{PI};

(d) the spread of place fields and positional inputs, σ;

(e) the number of neurons, N.

(f) the mean activity (fraction of active neurons at any time), a.

A fully-detailed analysis of the response of the system to the each of these parameters is beyond the scope of this paper, and previous works have fully characterized the behavior of the model in the absence of positional inputs [1–3]. Hereafter, we show how some of these parameters control the dynamical properties of the *flickering* of the cognitive map and the *ability to navigate*, i.e. the correct positioning of the bump of activity in the position defined by the V and PI inputs. These two quantities are indeed observable in the CA3 electrophysiology data, through the map and position-decoding analysis. A characterization of their parametric dependence in the model is therefore a necessary step to a correct quantitative modelling.

For this reason, we will here divide the parameters into two classes:

- the *structural* parameters, N, σ, a. The number of neurons N was varied from a few hundreds to a few thoousands in simulations. To keep the contributions to the total in put $H_{i,t}$ acting on neuron i at time t independent of N, we scale the recurrent connection strength γ_J as $1/N$, see Eqn. (9) in Main Text. This ensures that the sum of local inputs over all active neurons due to these connections has a finite, fixed value as N grows. This is why we will compare below the value of $\gamma_J \times N$ to the other input strengths, γ_V and γ_{PI}. In addition, we have fixed the average linear size of place fields to $\sigma/L \sim 0.125$, which sets the average area occupied by a place field to $2\pi(\sigma/L)^2 \simeq 10\%$ of the environment total area, a value comparable to experimental findings [4]. The average activity (in a time bin) was fixed to $a = 10\%$ throughout our simulations to match the values fixed in previous works focusing on the same model in the absence of inputs, see discussions in [2, 3].

- the *control* parameters (γ_J, γ_V, γ_{PI}, β), that have a predictable influence on the behaviors we are interested in. Note that the four control parameters are redundant, as the properties of the model depend only on $(\beta \times \gamma_J, \beta \times \gamma_{PI}, \beta \times \gamma_V)$; we may therefore fix one of them and let the other three vary. We now study how the model properties depend on the values of these parameters.

Navigation of the environment

The model is explicitly designed to mimic the representation of self-location in the hippocampal network under the influence of positional inputs. A natural question is how the values of parameters

influence the capability of the model to actually represent the correct position in a single map, that is, the correct centering of the neural bump around the input position. Consider the case of coherent inputs at a certain time t, i.e. PI and V point to the same position $\mathbf{r}_t$ in the same map , and let us assume that the bump is correctly centered around $\mathbf{r}_t$. As the input position changes in the next simulated time bin $t + \Delta t$, PI and V will try to activate place cells corresponding to a shifted location, effectively pushing the bump to $\mathbf{r}_{t+\Delta t}$. If the positional input is too weak compared to the recurrent network connections are too strong, the bump will fail to update to the new position, being trapped by the strong connection with the active cells at position $\mathbf{r}_t$. Similarly, a very high value of β, i.e. a low neural noise, would have the effect of enhancing the roughness of the energy landscape, in the positional space, and of trapping the bump and imparing its motion. As a consequence, the model would lose the ability to correctly navigate the environment. Conversely, a very low value of β would result in the inability of the model to condensate the bump of activity [3], therefore losing any notion of represented position.

The inverse of the mean positional error ϵ_t can be used as a proxy for the navigation ability, and is shown in Supplementary Fig. B as a function of β and of the relative strength $\gamma_V/(N\gamma_J)$ (in the balanced case $\gamma_V = \gamma_{PI}$). The navigable region (yellow) has a triangular shape that widens with higher values of the input strength, meaning that the temperature has to be fine tuned for low values of $\gamma_{V/PI}$, while it can take a wider set of values in the presence of strong inputs.

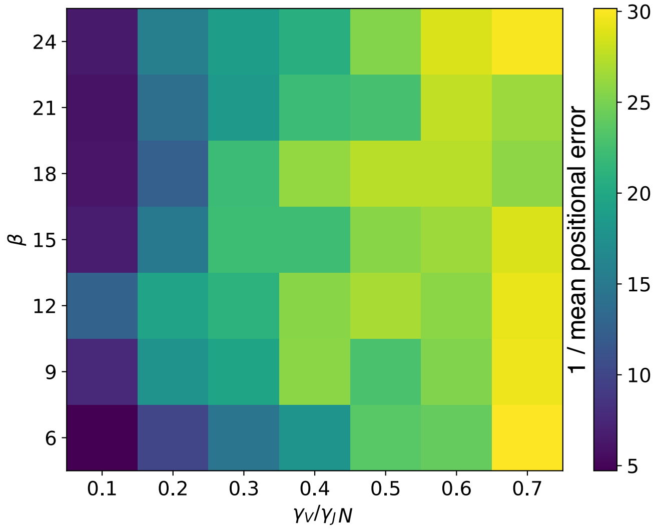

Supplementary Figure B. Navigation of the environment. Dependence of the inverse mean positional error (in cm^{-1}) on the control parameters: β and the relative strength between network connectivity and positional inputs. $\gamma_V = \gamma_{PI}$, $N = 400$, $a = 0.1$.

Flickering of the cognitive map

As discussed above, the system acts as an effective two-state model when the two inputs are put into conflict, i.e. point to the same position in different cognitive maps. The transition of the bump from one map to the other happens stochastically, and its dynamical properties are controlled by the

parameters of the model. Characterizing this dynamics in the simulated test experiment is rather involved, since the positional inputs move at a variable speed (we use the recorded trajectory of the real rat as input) and a fast change of the positional input can facilitate the evaporation of the bump from one map, increasing the transition rate between maps. We here analyze the dependence of two data-testable quantities on the parameters. The first is the statistics of permanence in the visual-cue associated map or in the PI-associated map during the conflicting phase, as a function of the relative strength between the two inputs, shown in Supplementary Fig. C. We see that a ration γ_V/γ_{PI} close to 1 results in a mean fraction of flickers (MFF) close to 0.55. This value, slightly different from the expected 0.5 is due to the inertia of the bump that, for few bins after the teleportation, tends to stay in the PI-associated map. Since each simulation is carried for a finite number of time bins after the teleportation (600), this discrepancy is explained as a consequence of the finite-time simulated for each trial.

Next we analyze a dynamical quantity, i.e. the mean sojourn time of the activity in one of the two maps, given a balanced value of $\gamma_{PI} = \gamma_V$, see Methods for the definition of the sojourn time. This quantity is directly proportional to the height of the barrier described in the two-state approximation, which is controlled by the network connectivity strength γ_J and the parameter β. High noise (small β) or weak connections (low γ_J) is expected to enhance the probabiity of crossing the barrier easy, and to make the sojourn times low. This statement is confirmed by the results shown in the diagram in Supplementary Fig. D.

Putting together the results reported in Supplementary Figs. B and D, we see that the model reproduces the dynamical properties of the observed data, while at the same time keeping an accurate representation of the input position, for a range of parameters in the center of the diagrams. In particular, we have chosen, for the simulations reported in the Main Text, $\beta = 15$ and $\gamma_V = \gamma_{PI} = 0.4$, with no need for fine tuning these two parameters. Indeed any choice in the range $\beta \in [15, 30]$ and $\gamma_V = \gamma_{PI} \in [0.3, 0.6]$ would qualitatively reproduce the behavior observed in data in terms of flickering of the cognitive map and precision in the positional encoding.

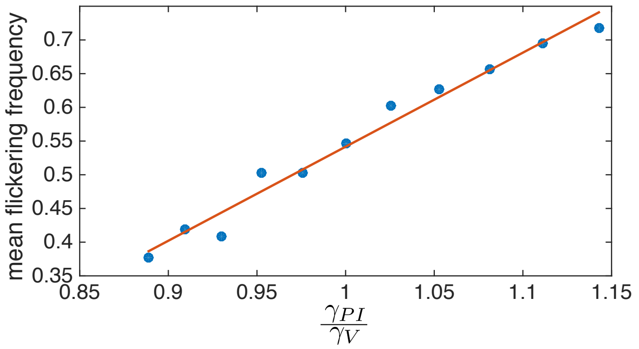

Supplementary Figure C. Dependence of the mean frequency of flickers (MFF) in simulated data upon model parameters. The MFF is defined as the fraction of time bins, during the conflicting phase, in which the hippocampal representation m differs from light cues. Simulations were performed using the real trajectory of the rat, with $N = 400$ neurons, $\gamma_W \sim \infty$ to hold the conflicting state to a fixed amount of time bins (600), $\beta = 15$.

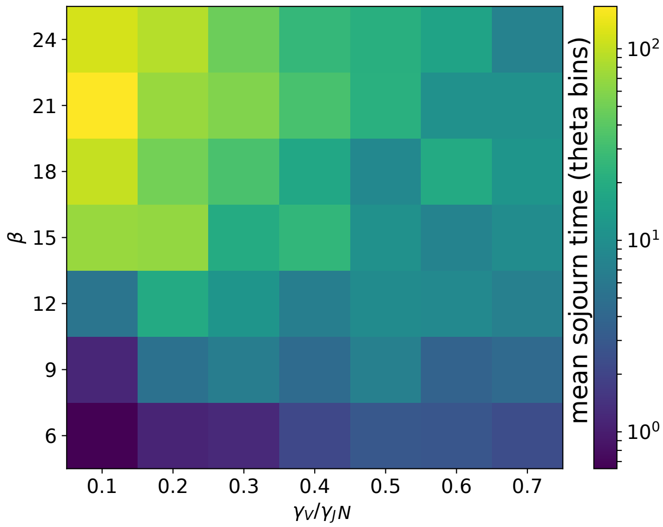

Supplementary Figure D. Flickering behavior: Dependence of the mean sojourn time, defined as the number of consecutive theta bin in which the bump is condensed in the same map, from the control parameters: β and the relative strength between network connectivity and positional inputs. $\gamma_V = \gamma_{PI}$, $N = 400$, $a = 0.1$.

3 Relationship between sojourn time and correlation time

The correlation time τ_0 (see Main Text Methods and Main Text Fig. 2B & 4C) is related to the sojourn times of the neural bump in the cognitive maps, defined as a sequence of contiguous theta bins that are all decoded in the same map. The relationship between correlation and sojourn time can be established by assuming a Markovian dynamics for the 2-state model ($m = A$ or $m = B$) evolving in discrete time. The dynamics is determined by the map transition probabilities from one time bin to the next:

$$\begin{cases} p_{A\to A} = e^{-1/\tau_A} \\ p_{A\to B} = 1 - e^{-1/\tau_A} \\ p_{B\to B} = e^{-1/\tau_B} \\ p_{B\to A} = 1 - e^{-1/\tau_B} \end{cases} \tag{7}$$

where τ_A and τ_B are the mean sojourn times in, respectively, map A and B. A straightforward calculation shows that the time correlation $C(\tau)$ between the map state at times t and $t+\tau$ is decreases exponentially with the delay τ only, with an average time equal to

$$\tau_0 = -\left[\log\left(e^{-1/\tau_A} + e^{-1/\tau_B} - 1\right)\right]^{-1} . \tag{8}$$

Hence, the correlation time τ_0 is approximately given by the smaller mean sojourn time among τ_A and τ_B. The distribution of sojourn times in each map for experimental CA3 data [5] is shown in Supplementary Fig. E.

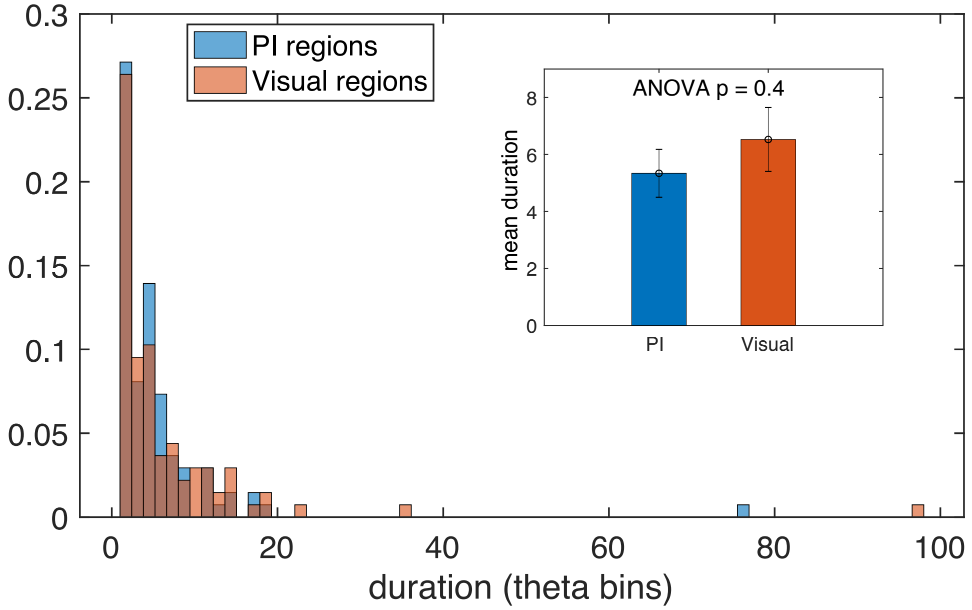

Supplementary Figure E. Sojourn times of hippocampal activity in the two cognitive maps during the conflicting phase for CA3 recordings. Regions of consecutive theta bins whose decoded representation disagree with the external light conditions are marked as "PI" regions. Vicevesa, if they agree with light conditions, they are marked as "Visual". Results obtained after application of our map decoder to the recorded CA3 data of [5]. As shown in the bar plot and from the ANOVA comparison, the permanence times in the two maps during the conflicting phase have roughly the same distribution. Results obtained with map-decoding threshold $L_0 = 2.3$.

4 Inference of path-integrator realignment times - discussion on parameters p_0 and p_e

To identify the realignment times of the PI we first introduce a simple probabilistic model for the hippocampal representation to be incoherent with the light-cue conditions (flickering time bin) as a function of time elapsed after the switch (see Main Text Methods). This procedure needs an input value for p_0, the probability of flickering during the conflicting phase, whose consistency can be checked a posteriori by computing the mean flickering frequency in the conflicting phase. In Supplementary Fig. F we show that the a-posteriori average flickering frequency remains remarkably stable, around the self-consistent choice ~ 0.6, for any input value of p_0, with a slight dependence on the chosen L_0 threshold. The same value is observed in the model when the strength of PI and V projections are set to similar values ($\gamma_{PI}/\gamma_V \in [0.95, 1.05]$), see Supplementary Fig. C.

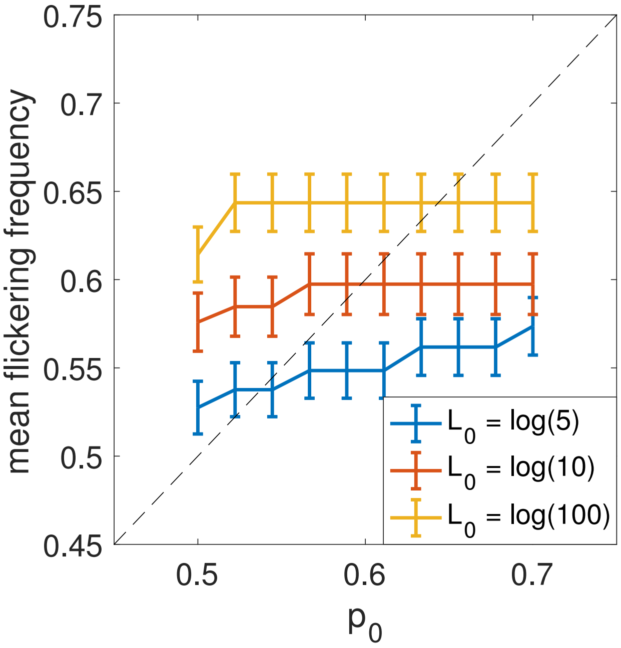

Supplementary Figure F. Dependence of mean frequency of flickers in recorded data upon threshold L_0 and upon the mean frequency p_0 used in the inference of PI-realignment time. A self-consistent value of p_0 is identified, for each value of map-decoding statistical threshold L_0, as the intersection between the corresponding curve and the $y = x$ line (Methods). Simulations performed with the same parameters described in Fig. 2, main text.

5 Independence of frequency of flickers from delay after light switch: parameters p_0 and p_e and L_0

As pointed in the main text, the constant flickering frequency hypothesis $H_{constant}$ is extremely more likely ($\Delta\ell \sim 150$) than the decaying model H_{decay}. The result is robust against changes in the parameters, see Supplementary Fig. F. For instance, we obtain $\Delta\ell \simeq 170$ and $\Delta\ell \simeq 60$ when the flickering identification is done based on, respectively, a less ($L_0 = \log 2$) and more ($L_0 = \log 100$) restrictive criterion. Similarly, the log-likelihood difference between the two hypothesis remains very large and positive if we change the constant-rate model p_0 value, e.g. $\Delta\ell \simeq 160$ for $p_0 = 0.4$, $\Delta\ell \simeq 120, 33$ for $p_e = 0.1, 0.001$, or if we extend the definition of H_{decay} up to 30 seconds after the switch (instead of 15 seconds): $\Delta\ell \simeq 125$. In many teleportation events the flickering-dense area is indeed too short (too few flickers) or too long (too many flickers occurring far away the teleportation time) to be explained by the H_{decay} hypothesis, see Supplementary Fig. G.

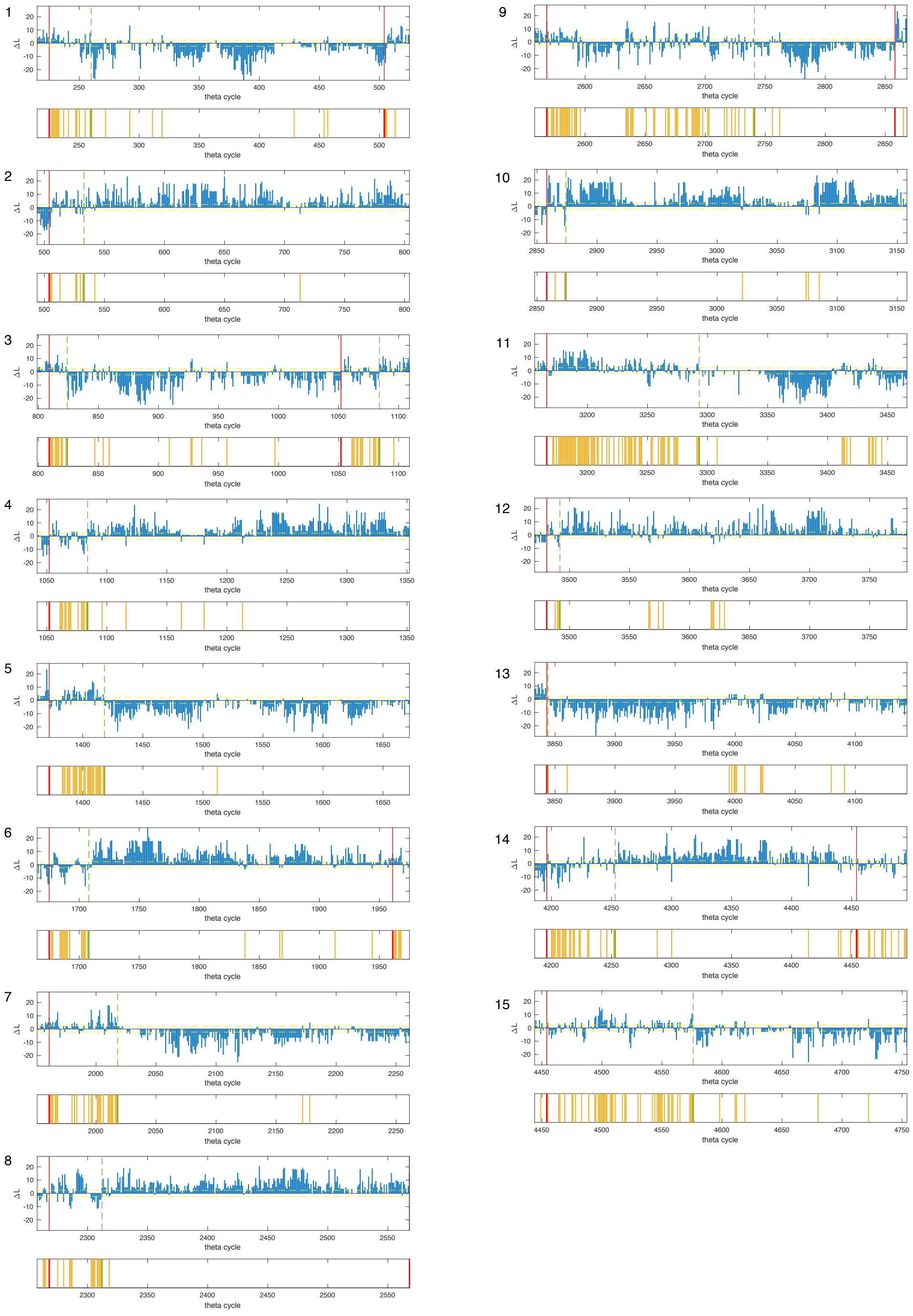

Supplementary Figure G. Decoded maps as a function of time for all 15 **light-switch events in the test session**. Same analysis in main text Fig. 1C, which was restricted to a single light switch. PI-realignment times are marked with a dashed green line.

6 Assessment of performances of map decoder

Our map decoder (based on the inference of an Ising model for each cognitive map) does not use any information about the current rodent position. Its performance can be assessed against the correlation-based decoders used in [5] (which compares the activity $\mathbf{s}_t$ to the expected activities in maps A and B at the given rat position) by means of the classical binary-classifier theory [6–9]. The Ising model was shown, first on retinal ganglion cell recordings, and, more recently, on prefrontal cortex [10, 11] and hippocampal data [12, 13], to provide a good approximation for the distribution of population activity configurations. The performance in the decoding task has been shown to be superior to rate-based decoders on CA1 data [12].

The standard tool used to compute the performance of binary decoders is the Receiver Operating Characteristic (ROC) diagram [6]. This diagram is drawn by computing the true positive rate (TPR) and false positive rate (FPR) as a function of different thresholding values, and plotting the resulting curve in the TPR-FPR plane. These quantities can be defined in the context of our map decoder as follows: for each theta bin t the decoder outputs a value $\Delta\mathcal{L}(t)$, which is then interpreted as referring to map A or B depending on its value compared to a moving threshold Θ. Note that this is slightly different from the map-decoding method reported in Main Text, since it does not allow undecoded statistically-not-significant bins.

$$m_t = \begin{cases} A & if \;\; \Delta\mathcal{L}(t) > \Theta \;, \\ B & if \;\; \Delta\mathcal{L}(t) < \Theta \;. \end{cases} \tag{9}$$

To match the vocables used in the ROC framework we will arbitrarily follow the convention that the output is positive if the map is decoded to be A, and negative if the map is predicted to be B. Doing so, a True Positive is defined as a correctly-decoded environment A (with respect to the light conditions: $m_t = A =$ light cues), while a True Negative will be a correctly-decoded environment B. The final observable (area under the ROC curve) is symmetrical under the inversion of this convention, which is summarized in Table 1. The decoding capability is finally assessed by applying the decoder to two "constant" test sessions, where the environment is constantly set to A and B, respectively. Assuming that the neural representation is stable under fixed light conditions, we can compute the TPR and FPR of the decoder by counting how many theta bins are correctly and falsely decoded in the two reference sessions. For a specific value of the threshold Θ, this corresponds to a point in the FPR-TPR plane. By varying this value we then draw the curve as the succession of the corresponding TPR-FPR values. The standard quantitative measure of the decoding performances is the Area Under the Curve (AUC) of the ROC diagram [6]. According to this measure, the ideal decoder has AUC = 1, while random guessing would give AUC = 0.5. All the decoders, tested on constant test sessions, i.e. where no teleportation is performed, show very high performances, see Supplementary Fig. H. Note, in addition, that our functional-network based decoder is robust against the presence of correlations between the maps: it shows much better performance than correlation-based methods for CA1 recordings, where maps are much less orthogonal than in CA3 [12].

7 Dependence of positional-error analysis with L_0

The significance of positional-error analysis as a function of the threshold L_0 is shown in Supplementary Fig. I.

decoder output	A	B	A	B
light conditions	A	A	B	B
denomination	True Positive	False Negative	False Positive	True Negative

Table 1: Denominations used for the four possible events, depending on the output of the decoder and on the environment-defining cue. The cue is not changed throughout the reference session.

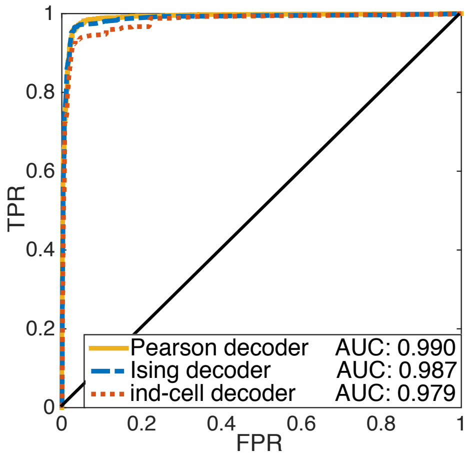

Supplementary Figure H. ROC curves for our Ising-model map decoder (blue) compared to independent-cell decoder (specific case of Ising model, with zero couplings J, red dotted curve) and the Pearson-correlation based decoder of [5], which used the true position of the rodent.

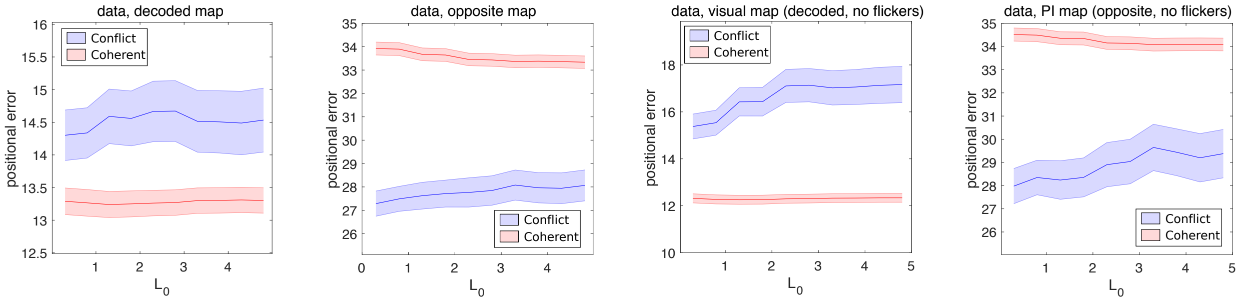

Supplementary Figure I. Significance of positional-error analysis as a function L_0 Mean positional error (line) and standard error (shaded area) for CA3 data reported in main text Figure 6 A (position inferred according to the decoded map), Figure 6 B (position inferred according to the opposite map) and Figure 6 C,D (position inferred according to the decoded/opposite map without considering flickering events), for a large range of values of the threshold L_0 of the map decoder used in the conflict period identification; higher values of L_0 correspond to shorter decoded conflict periods.

References

[1] R. Monasson and S. Rosay, "Crosstalk and transitions between multiple spatial maps in an attractor neural network model of the hippocampus: Collective motion of the activity," Physical review E, vol. 89, no. 3, p. 032803, 2014.

[2] R. Monasson and S. Rosay, "Crosstalk and transitions between multiple spatial maps in an attractor neural network model of the hippocampus: Phase diagram," Physical review E, vol. 87, no. 6, p. 062813, 2013.

[3] R. Monasson and S. Rosay, "Transitions between spatial attractors in place-cell models," Physical review letters, vol. 115, no. 9, p. 098101, 2015.

[4] S. A. Hollup, S. Morlen, J. G. Donnett, M.-B. Moser, and E. I. Moser, "Accumulation of hippocampal place fields at the goal location in an annular watermaze task," The Journal of Neuroscience, vol. 21, pp. 1635–1644, 2001.

[5] K. Jezek, E. J. Henriksen, A. Treves, E. I. Moser, and M.-B. Moser, "Theta-paced flickering between place-cell maps in the hippocampus," Nature, vol. 478, no. 7368, pp. 246–249, 2011.

[6] J. A. Hanley and B. J. McNeil, "The meaning and use of the area under a receiver operating characteristic (roc) curve.," Radiology, vol. 143, no. 1, pp. 29–36, 1982.

[7] C. E. Metz, "Basic principles of roc analysis," in Seminars in nuclear medicine, vol. 8, pp. 283–298, Elsevier, 1978.

[8] A. P. Bradley, "The use of the area under the roc curve in the evaluation of machine learning algorithms," Pattern recognition, vol. 30, no. 7, pp. 1145–1159, 1997.

[9] M. J. Pencina, R. B. D'Agostino, R. B. D'Agostino, and R. S. Vasan, "Evaluating the added predictive ability of a new marker: from area under the roc curve to reclassification and beyond," Statistics in medicine, vol. 27, no. 2, p. 157, 2008.

[10] G. Tavoni, S. Cocco, and R. Monasson, "Neural assemblies revealed by inferred connectivity-based models of prefrontal cortex recordings," Journal of computational neuroscience, vol. 41, no. 3, pp. 269–293, 2016.

[11] G. Tavoni, U. Ferrari, F. P. Battaglia, S. Cocco, and R. Monasson, "Functional coupling networks inferred from prefrontal cortex activity show experience-related effective plasticity," Network Neuroscience, vol. 1, no. 3, pp. 275–301, 2017.

[12] L. Posani, S. Cocco, K. Ježek, and R. Monasson, "Functional connectivity models for decoding of spatial representations from hippocampal ca1 recordings," Journal of Computational Neuroscience, pp. 1–17, 2017.

[13] L. Meshulam, J. L. Gauthier, C. D. Brody, D. W. Tank, and W. Bialek, "Collective behavior of place and non-place neurons in the hippocampal network," Neuron, vol. 96, no. 2, pp. 1178–1191, 2017.
